# 2-[*N*-(4-Meth­oxy­phen­yl)acetamido]-1,3-thia­zol-4-yl acetate

**DOI:** 10.1107/S1600536813004236

**Published:** 2013-02-16

**Authors:** Volodymyr Horishny, Roman Lesyk, Andrzej K. Gzella

**Affiliations:** a Department of Pharmaceutical, Organic and Bioorganic Chemistry, Danylo Halytsky Lviv National Medical University, Pekarska 69, Lviv, 79010, Ukraine; b Department of Organic Chemistry, Poznan University of Medical Sciences, ul. Grunwaldzka 6, 60-780 Poznań, Poland; c Faculty of Pharmacy, Ludwik Rydygier Collegium Medicum in Bydgoszcz, Nicolaus Copernicus University in Torun, ul. A. Jurasza 2, 85-089 Bydgoszcz, Poland

## Abstract

The structural analysis of the title compound, C_14_H_14_N_2_O_4_S, particularly the presence of an acetyl group at the exocyclic N atom and the C(H)—C(O_2_CMe)—N acet­oxy group in the thia­zole ring, may indicate that one of the starting materials, *i.e.* 2-(4-meth­oxy­anilino)-1,3-thia­zol-4(5*H*)-one, exists in the reaction mixture, at least partially, as a tautomer with an exocyclic amine N atom and an enol group. The acet­oxy and acetyl groups deviate from the thia­zole plane by 69.17 (6) and 7.25 (19)°, respectively. The thia­zole and benzene rings form a dihedral angle of 73.50 (4)°. In the crystal, centrosymmetrically related mol­ecules are connected into dimeric aggregates *via* C—H⋯O inter­actions.

## Related literature

For the biological activity of 2-ar­yl(heter­yl)amino­thia­zol-4-one derivatives, see: Ates *et al.* (2000 ▶); Eleftheriou *et al.* (2012 ▶); Eriksson *et al.* (2007 ▶); Lesyk & Zimenkovsky (2004 ▶); Lesyk *et al.* (2003 ▶, 2011 ▶); Rout & Mahapatra (1955 ▶); Subtel’na *et al.* (2010 ▶). For prototropic tautomerism studies, see: Lesyk *et al.* (2003 ▶); Subtel’na *et al.* (2010 ▶). For bond-length data, see: Allen *et al.* (1987 ▶). For a related structural study, see: Horishny *et al.* (2013 ▶).
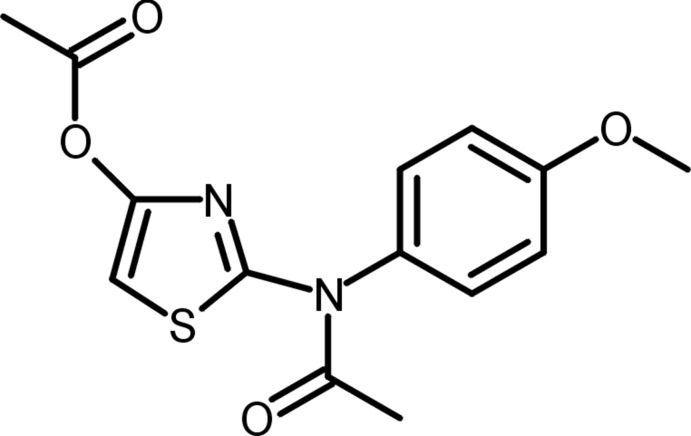


## Experimental

### 

#### Crystal data

C_14_H_14_N_2_O_4_S
*M* *_r_* = 306.33
Triclinic, 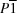

*a* = 8.9445 (5) Å
*b* = 9.5736 (8) Å
*c* = 9.9078 (9) Å
α = 115.509 (9)°
β = 93.381 (6)°
γ = 108.144 (6)°
*V* = 708.95 (10) Å^3^
*Z* = 2
Mo *K*α radiation
μ = 0.25 mm^−1^
*T* = 130 K
0.50 × 0.50 × 0.10 mm

#### Data collection

Agilent Xcalibur Atlas diffractometer
Absorption correction: multi-scan (*CrysAlis PRO*; Agilent, 2011 ▶) *T*_min_ = 0.860, *T*_max_ = 1.000
12469 measured reflections
3445 independent reflections
3075 reflections with *I* > 2σ(*I*)
*R*_int_ = 0.022

#### Refinement

*R*[*F* ^2^ > 2σ(*F* ^2^)] = 0.035
*wR*(*F* ^2^) = 0.095
*S* = 1.06
3445 reflections
193 parameters
H-atom parameters constrained
Δρ_max_ = 0.37 e Å^−3^
Δρ_min_ = −0.27 e Å^−3^

### 

Data collection: *CrysAlis PRO* (Agilent, 2011 ▶); cell refinement: *CrysAlis PRO*; data reduction: *CrysAlis PRO*; program(s) used to solve structure: *SHELXS97* (Sheldrick, 2008 ▶); program(s) used to refine structure: *SHELXL97* (Sheldrick, 2008 ▶); molecular graphics: *ORTEP-3 for Windows* (Farrugia, 2012 ▶); software used to prepare material for publication: *WinGX* (Farrugia, 2012 ▶) and *PLATON* (Spek, 2009 ▶).

## Supplementary Material

Crystal structure: contains datablock(s) I, global. DOI: 10.1107/S1600536813004236/tk5195sup1.cif

Structure factors: contains datablock(s) I. DOI: 10.1107/S1600536813004236/tk5195Isup2.hkl

Supplementary material file. DOI: 10.1107/S1600536813004236/tk5195Isup3.cml

Additional supplementary materials:  crystallographic information; 3D view; checkCIF report

## Figures and Tables

**Table 1:** Hydrogen-bond geometry (Å, °)

*D*—H⋯*A*	*D*—H	H⋯*A*	*D*⋯*A*	*D*—H⋯*A*
C5—H5⋯O20^i^	0.93	2.53	3.200 (2)	129

Symmetry code: (i) 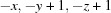.

**Fig. 1.**
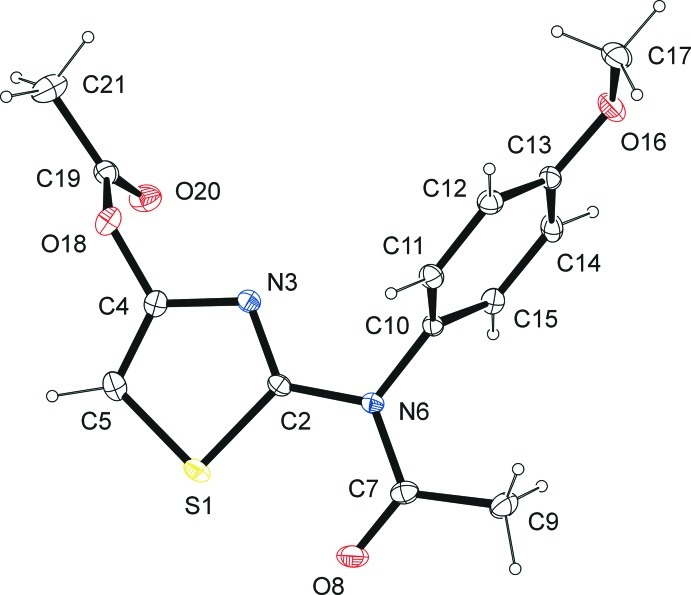
The molecular structure of (I) together with the atom-numbering scheme. Displacement ellipsoids are drawn at the 30% probability level and H atoms are shown as spheres of arbitrary radii.

**Fig. 2.**
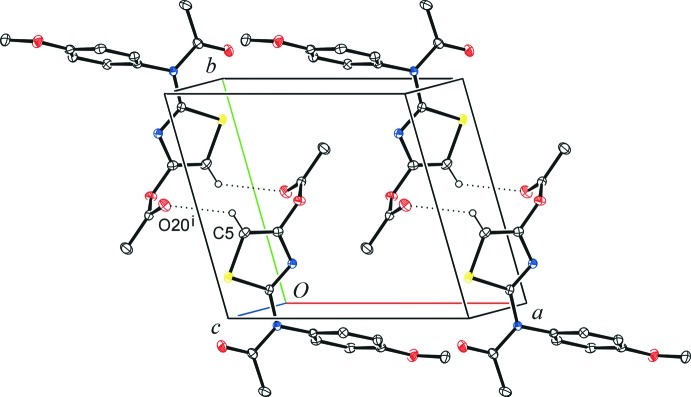
The hydrogen bonding (dotted lines) in the title structure. Symmetry code: (i) -x,1 - y,1 - z. H atoms not involved in hydrogen bonds have been omitted for clarity.

## supplementary crystallographic information

### Comment

Significant popularity of thiazolidine scaffolds in drug design is grounded on the broad spectrum of biological activity of their derivatives. Among thiazolidine derivatives the group of 2-aryl(heteryl)aminothiazol-4-one derivatives is one of the most promising (Lesyk & Zimenkovsky, 2004; Lesyk *et al.*, 2011). 2-Aryl(heteryl)aminothiazol-4-one activities covers antibacterial (Ates *et al.*, 2000), antifungal (Rout & Mahapatra, 1955), anti-inflammatory (Lesyk *et al.*, 2003; Eleftheriou *et al.*, 2012) and anticancer activities (Subtel'na *et al.*, 2010; Eriksson *et al.*, 2007). Moreover, literature reports indicate existence of prototropic tautomeric forms of 3-unsubstituted 2-aryl(heteryl)aminothiazol-4-ones both in solutions and solid phase which can be of significant importance for biological activity (Lesyk *et al.*, 2003; Subtel'na *et al.*, 2010). Dictated by these observations, the aim of the presented work was synthesis of the compound (I) as starting substance for further design of new biologically active compounds.

The investigations on the structure of the title compound, a product of the reaction of 2-(4-methoxyanilino)-1,3-thiazol-4-one with acetyl anhydride, showed the presence of an acetoxy group at C4 and an acetyl functionality at N6 (Fig. 1). Similar observations were made for the product obtained by the identical method from 2-(2,4-dimethoxyanilino)-1,3-thiazol-4-one. The presence of the C4 acetoxy and N6 acetyl groups in the structure of compound (I) and 2-[N-(2,4-dimethoxyphenyl)acetamido]-1,3-thiazol-4-yl acetate (Horishny *et al.*, 2013) may indicate that the starting materials, *i.e.* 2-(4-methoxyanilino)-1,3-thiazol-4-one and 2-(2,4-dimethoxyanilino)-1,3-thiazol-4-one, exist in the reaction mixture at least partially as tautomers with an exocyclic amine nitrogen and an enol moiety (*H—*)C5═C4—OH within the five-membered heterocyclic ring.

The C4 acetoxy group and N6 acetyl functionality are oriented differently in relation to the planar thiazole ring. The first one forms a dihedral angle of 79.22 (5)° with the mean plane of this ring whereas the second one is tilted only slightly [dihedral angle: 7.25 (19)°] (Fig. 1).

Both the C7═O8 carbonyl group relative to the C2—N6 bond and the C21═ O22 carbonyl group in relation to the C4—O20 bond have the same synperiplanar conformation. The torsional angles C2—N6—C7—O8 and C4—O18—C19—O20 are 4.96 (19) and -1.67 (19)°, respectively. The C13 methoxy group is approximately coplanar with the phenyl ring – the torsion angle is 1.9 (2)°. The flat phenyl and thiazole rings form a dihedral angle of 73.50 (4)°.

The bond lengths and angles in compound (I) are similar to those observed in 2-[*N*-(2,4-dimethoxyphenyl)acetamido]-1,3-thiazol-4-yl acetate (Horishny *et al.*, 2013). The N6—C7 distance [1.3876 (16) Å] is longer (by about 8σ) than the normal (*O*═)C—N tertiary amide distance [1.346 (5) Å, Allen *et al.*, 1987].

In the crystal structure, the molecules of (I) are connected by the C5—H5···O21^i^ hydrogen bonds into centrosymmetric dimers (Table 1, Fig. 2).

### Experimental

2-(4-Methoxyanilino)thiazol-4-one in the medium of acetic anhydride was refluxed for 2 h. The obtained solution was evaporated in vacuum and the residue was recrystallized twice from benzene–hexane (1:1) mixtures.

### Refinement

All H atoms were located into the idealized positions and were refined within the riding model approximation: C_methyl_—H = 0.96 Å, C(*sp*^2^)—H = 0.93 Å; *U*_iso_(H) = 1.2*U*_eq_(C) or 1.5*U*_eq_(C) for methyl H. The methyl groups were refined as rigid groups which were allowed to rotate.

### Crystal data

**Table:**

C_14_H_14_N_2_O_4_S	*Z* = 2
*M**_r_* = 306.33	*F*(000) = 320
Triclinic, *P*1	*D*_x_ = 1.435 Mg m^−^^3^
Hall symbol: -P 1	Melting point = 399–401 K
*a* = 8.9445 (5) Å	Mo *K*α radiation, λ = 0.71073 Å
*b* = 9.5736 (8) Å	Cell parameters from 5310 reflections
*c* = 9.9078 (9) Å	θ = 2.3–29.1°
α = 115.509 (9)°	µ = 0.25 mm^−^^1^
β = 93.381 (6)°	*T* = 130 K
γ = 108.144 (6)°	Block, yellow
*V* = 708.95 (10) Å^3^	0.50 × 0.50 × 0.10 mm

### Data collection

**Table:**

Agilent Xcalibur Atlas diffractometer	3445 independent reflections
Radiation source: fine-focus sealed tube	3075 reflections with *I* > 2σ(*I*)
Graphite monochromator	*R*_int_ = 0.022
Detector resolution: 10.3088 pixels mm^-1^	θ_max_ = 29.1°, θ_min_ = 2.3°
ω scans	*h* = −11→11
Absorption correction: multi-scan (*CrysAlis PRO*; Agilent, 2011)	*k* = −13→12
*T*_min_ = 0.860, *T*_max_ = 1.000	*l* = −12→13
12469 measured reflections	

### Refinement

**Table:**

Refinement on *F*^2^	Primary atom site location: structure-invariant direct methods
Least-squares matrix: full	Secondary atom site location: difference Fourier map
*R*[*F*^2^ > 2σ(*F*^2^)] = 0.035	Hydrogen site location: inferred from neighbouring sites
*wR*(*F*^2^) = 0.095	H-atom parameters constrained
*S* = 1.06	*w* = 1/[σ^2^(*F*_o_^2^) + (0.045*P*)^2^ + 0.2987*P*] where *P* = (*F*_o_^2^ + 2*F*_c_^2^)/3
3445 reflections	(Δ/σ)_max_ < 0.001
193 parameters	Δρ_max_ = 0.37 e Å^−^^3^
0 restraints	Δρ_min_ = −0.27 e Å^−^^3^

### Special details

**Table:**

Geometry. All e.s.d.'s (except the e.s.d. in the dihedral angle between two l.s. planes) are estimated using the full covariance matrix. The cell e.s.d.'s are taken into account individually in the estimation of e.s.d.'s in distances, angles and torsion angles; correlations between e.s.d.'s in cell parameters are only used when they are defined by crystal symmetry. An approximate (isotropic) treatment of cell e.s.d.'s is used for estimating e.s.d.'s involving l.s. planes.
Refinement. Refinement of *F*^2^ against ALL reflections. The weighted *R*-factor *wR* and goodness of fit *S* are based on *F*^2^, conventional *R*-factors *R* are based on *F*, with *F* set to zero for negative *F*^2^. The threshold expression of *F*^2^ > σ(*F*^2^) is used only for calculating *R*-factors(gt) *etc*. and is not relevant to the choice of reflections for refinement. *R*-factors based on *F*^2^ are statistically about twice as large as those based on *F*, and *R*- factors based on ALL data will be even larger.

### Fractional atomic coordinates and isotropic or equivalent isotropic displacement parameters (Å^2^)

**Table:**

	*x*	*y*	*z*	*U*_iso_*/*U*_eq_	
S1	−0.09020 (4)	0.14762 (4)	0.45516 (4)	0.02269 (10)	
C2	0.03052 (15)	0.08180 (16)	0.32947 (14)	0.0177 (2)	
N3	0.16077 (13)	0.19958 (13)	0.34043 (12)	0.0191 (2)	
C4	0.16774 (16)	0.34906 (16)	0.45435 (15)	0.0215 (3)	
C5	0.04751 (17)	0.34910 (17)	0.52956 (16)	0.0249 (3)	
H5	0.0399	0.4428	0.6090	0.030*	
N6	−0.00495 (12)	−0.08552 (13)	0.22511 (12)	0.0182 (2)	
C7	−0.14419 (16)	−0.20995 (17)	0.21360 (16)	0.0233 (3)	
O8	−0.24272 (12)	−0.17289 (13)	0.28765 (13)	0.0308 (2)	
C9	−0.16476 (18)	−0.38706 (18)	0.10871 (18)	0.0305 (3)	
H9A	−0.2535	−0.4615	0.1245	0.046*	
H9B	−0.0678	−0.4040	0.1305	0.046*	
H9C	−0.1861	−0.4089	0.0040	0.046*	
C10	0.11162 (15)	−0.12397 (15)	0.13403 (14)	0.0182 (2)	
C11	0.25487 (16)	−0.11824 (17)	0.20057 (15)	0.0220 (3)	
H11	0.2770	−0.0887	0.3042	0.026*	
C12	0.36650 (16)	−0.15662 (17)	0.11261 (16)	0.0237 (3)	
H12	0.4633	−0.1527	0.1569	0.028*	
C13	0.33070 (16)	−0.20089 (16)	−0.04265 (16)	0.0229 (3)	
C14	0.18573 (17)	−0.20726 (17)	−0.10887 (15)	0.0240 (3)	
H14	0.1623	−0.2383	−0.2128	0.029*	
C15	0.07619 (16)	−0.16761 (16)	−0.02071 (15)	0.0213 (3)	
H15	−0.0201	−0.1701	−0.0645	0.026*	
O16	0.42891 (13)	−0.24239 (14)	−0.14095 (12)	0.0321 (2)	
C17	0.57663 (19)	−0.2456 (2)	−0.0825 (2)	0.0358 (4)	
H17A	0.6389	−0.1390	0.0039	0.054*	
H17B	0.6368	−0.2696	−0.1611	0.054*	
H17C	0.5537	−0.3304	−0.0509	0.054*	
O18	0.30433 (12)	0.49009 (12)	0.49350 (11)	0.0263 (2)	
C19	0.31110 (16)	0.56737 (17)	0.40442 (16)	0.0238 (3)	
O20	0.20654 (12)	0.51567 (14)	0.29430 (12)	0.0311 (2)	
C21	0.46079 (18)	0.7203 (2)	0.4657 (2)	0.0372 (4)	
H21A	0.5421	0.6945	0.4116	0.056*	
H21B	0.4991	0.7596	0.5730	0.056*	
H21C	0.4376	0.8053	0.4520	0.056*	

### Atomic displacement parameters (Å^2^)

**Table:**

	*U*^11^	*U*^22^	*U*^33^	*U*^12^	*U*^13^	*U*^23^
S1	0.02568 (18)	0.02852 (19)	0.02403 (18)	0.01508 (14)	0.01369 (13)	0.01672 (15)
C2	0.0191 (6)	0.0226 (6)	0.0167 (6)	0.0101 (5)	0.0062 (4)	0.0118 (5)
N3	0.0194 (5)	0.0210 (5)	0.0186 (5)	0.0081 (4)	0.0053 (4)	0.0102 (4)
C4	0.0252 (6)	0.0201 (6)	0.0195 (6)	0.0085 (5)	0.0027 (5)	0.0098 (5)
C5	0.0336 (7)	0.0255 (7)	0.0211 (6)	0.0165 (6)	0.0092 (5)	0.0117 (6)
N6	0.0182 (5)	0.0195 (5)	0.0183 (5)	0.0067 (4)	0.0064 (4)	0.0100 (4)
C7	0.0215 (6)	0.0257 (7)	0.0246 (7)	0.0057 (5)	0.0047 (5)	0.0157 (6)
O8	0.0236 (5)	0.0334 (6)	0.0390 (6)	0.0084 (4)	0.0141 (4)	0.0207 (5)
C9	0.0312 (7)	0.0221 (7)	0.0329 (8)	0.0028 (6)	0.0072 (6)	0.0135 (6)
C10	0.0212 (6)	0.0159 (5)	0.0181 (6)	0.0069 (5)	0.0075 (5)	0.0084 (5)
C11	0.0238 (6)	0.0241 (6)	0.0188 (6)	0.0087 (5)	0.0057 (5)	0.0110 (5)
C12	0.0208 (6)	0.0247 (6)	0.0258 (7)	0.0086 (5)	0.0051 (5)	0.0122 (6)
C13	0.0286 (7)	0.0173 (6)	0.0235 (7)	0.0086 (5)	0.0122 (5)	0.0095 (5)
C14	0.0343 (7)	0.0217 (6)	0.0158 (6)	0.0108 (5)	0.0068 (5)	0.0084 (5)
C15	0.0249 (6)	0.0200 (6)	0.0196 (6)	0.0085 (5)	0.0036 (5)	0.0098 (5)
O16	0.0367 (6)	0.0362 (6)	0.0288 (5)	0.0192 (5)	0.0182 (4)	0.0148 (5)
C17	0.0318 (8)	0.0345 (8)	0.0471 (10)	0.0178 (7)	0.0216 (7)	0.0192 (7)
O18	0.0276 (5)	0.0202 (5)	0.0259 (5)	0.0053 (4)	0.0000 (4)	0.0097 (4)
C19	0.0227 (6)	0.0233 (6)	0.0278 (7)	0.0112 (5)	0.0095 (5)	0.0120 (6)
O20	0.0288 (5)	0.0357 (6)	0.0291 (5)	0.0071 (4)	0.0047 (4)	0.0194 (5)
C21	0.0253 (7)	0.0317 (8)	0.0537 (10)	0.0046 (6)	0.0024 (7)	0.0245 (8)

### Geometric parameters (Å, º)

**Table:**

S1—C5	1.7233 (15)	C11—H11	0.9300
S1—C2	1.7379 (12)	C12—C13	1.3944 (19)
C2—N3	1.3046 (16)	C12—H12	0.9300
C2—N6	1.3979 (17)	C13—O16	1.3649 (16)
N3—C4	1.3678 (17)	C13—C14	1.390 (2)
C4—C5	1.3439 (19)	C14—C15	1.3835 (18)
C4—O18	1.3899 (16)	C14—H14	0.9300
C5—H5	0.9300	C15—H15	0.9300
N6—C7	1.3876 (16)	O16—C17	1.4258 (19)
N6—C10	1.4494 (15)	C17—H17A	0.9600
C7—O8	1.2196 (17)	C17—H17B	0.9600
C7—C9	1.501 (2)	C17—H17C	0.9600
C9—H9A	0.9600	O18—C19	1.3677 (17)
C9—H9B	0.9600	C19—O20	1.1984 (17)
C9—H9C	0.9600	C19—C21	1.491 (2)
C10—C11	1.3801 (18)	C21—H21A	0.9600
C10—C15	1.3907 (18)	C21—H21B	0.9600
C11—C12	1.3953 (18)	C21—H21C	0.9600
			
C5—S1—C2	88.70 (6)	C13—C12—C11	119.01 (12)
N3—C2—N6	121.23 (11)	C13—C12—H12	120.5
N3—C2—S1	115.46 (10)	C11—C12—H12	120.5
N6—C2—S1	123.30 (9)	O16—C13—C14	114.88 (12)
C2—N3—C4	108.79 (11)	O16—C13—C12	124.64 (13)
C5—C4—N3	118.01 (12)	C14—C13—C12	120.48 (12)
C5—C4—O18	123.64 (12)	C15—C14—C13	120.23 (12)
N3—C4—O18	118.17 (11)	C15—C14—H14	119.9
C4—C5—S1	109.03 (10)	C13—C14—H14	119.9
C4—C5—H5	125.5	C14—C15—C10	119.31 (12)
S1—C5—H5	125.5	C14—C15—H15	120.3
C7—N6—C2	120.87 (11)	C10—C15—H15	120.3
C7—N6—C10	121.59 (11)	C13—O16—C17	117.97 (12)
C2—N6—C10	117.51 (10)	O16—C17—H17A	109.5
O8—C7—N6	119.90 (12)	O16—C17—H17B	109.5
O8—C7—C9	123.04 (12)	H17A—C17—H17B	109.5
N6—C7—C9	117.05 (12)	O16—C17—H17C	109.5
C7—C9—H9A	109.5	H17A—C17—H17C	109.5
C7—C9—H9B	109.5	H17B—C17—H17C	109.5
H9A—C9—H9B	109.5	C19—O18—C4	117.42 (10)
C7—C9—H9C	109.5	O20—C19—O18	122.46 (12)
H9A—C9—H9C	109.5	O20—C19—C21	126.78 (13)
H9B—C9—H9C	109.5	O18—C19—C21	110.75 (12)
C11—C10—C15	120.86 (12)	C19—C21—H21A	109.5
C11—C10—N6	120.11 (11)	C19—C21—H21B	109.5
C15—C10—N6	119.03 (11)	H21A—C21—H21B	109.5
C10—C11—C12	120.11 (12)	C19—C21—H21C	109.5
C10—C11—H11	119.9	H21A—C21—H21C	109.5
C12—C11—H11	119.9	H21B—C21—H21C	109.5
			
C5—S1—C2—N3	−0.81 (10)	C7—N6—C10—C15	75.64 (16)
C5—S1—C2—N6	177.79 (11)	C2—N6—C10—C15	−106.51 (13)
N6—C2—N3—C4	−177.87 (11)	C15—C10—C11—C12	−0.1 (2)
S1—C2—N3—C4	0.76 (13)	N6—C10—C11—C12	179.45 (11)
C2—N3—C4—C5	−0.29 (16)	C10—C11—C12—C13	−0.1 (2)
C2—N3—C4—O18	174.95 (10)	C11—C12—C13—O16	−179.44 (12)
N3—C4—C5—S1	−0.30 (15)	C11—C12—C13—C14	−0.2 (2)
O18—C4—C5—S1	−175.25 (10)	O16—C13—C14—C15	−179.91 (12)
C2—S1—C5—C4	0.58 (10)	C12—C13—C14—C15	0.8 (2)
N3—C2—N6—C7	−179.65 (11)	C13—C14—C15—C10	−1.00 (19)
S1—C2—N6—C7	1.83 (17)	C11—C10—C15—C14	0.63 (19)
N3—C2—N6—C10	2.48 (17)	N6—C10—C15—C14	−178.89 (11)
S1—C2—N6—C10	−176.04 (9)	C14—C13—O16—C17	−177.34 (12)
C2—N6—C7—O8	4.96 (19)	C12—C13—O16—C17	1.9 (2)
C10—N6—C7—O8	−177.26 (12)	C5—C4—O18—C19	−102.83 (15)
C2—N6—C7—C9	−174.65 (12)	N3—C4—O18—C19	82.22 (15)
C10—N6—C7—C9	3.14 (18)	C4—O18—C19—O20	−1.67 (19)
C7—N6—C10—C11	−103.88 (14)	C4—O18—C19—C21	177.30 (12)
C2—N6—C10—C11	73.97 (15)		

### Hydrogen-bond geometry (Å, º)

**Table:**

*D*—H···*A*	*D*—H	H···*A*	*D*···*A*	*D*—H···*A*
C5—H5···O20^i^	0.93	2.53	3.200 (2)	129

Symmetry code: (i) −*x*, −*y*+1, −*z*+1.
